# Continuous central venous saturation monitoring in critically ill patients

**DOI:** 10.1186/cc9459

**Published:** 2011-03-11

**Authors:** D Chiumello, M Cressoni, A Marino, E Gallazzi, C Mietto, V Berto, M Chierichetti

**Affiliations:** 1 Fondazione IRCCS Ca' Granda-Ospedale Maggiore Policlinico, Milan, Italy; 2 Università degli Studi di Milano, Milan, Italy

## Introduction

Central venous oxygen saturation (ScvO_2_) is a useful therapeutic target in septic shock. ScvO_2 _is an indirect index of the balance between oxygen supply and demand, thus in critically ill patients a fall in ScvO_2 _reflects a decrease in tissue oxygenation. ScvO_2 _depends on arterial oxygen saturation, oxygen consumption, cardiac output and hemoglobin. The aim of the study was to evaluate events of tissue oxygenation impairment that could be unrecognized by simple blood gas analysis, by continuously monitoring ScvO_2 _and to establish whether peripheral oxygen saturation (SpO_2_), mean arterial pressure (MAP), heart rate (HR), and central venous pressure (CVP) could predict LowScvO_2 _events.

## Methods

Ventilated critically ill patients requiring a central venous catheter (CVC) for clinical use were enrolled. Continuous ScvO_2 _monitoring was obtained by a fiberoptic sensor inserted in the CVC and recorded for 72 hours with SpO_2_, HR, MAP and CVP. LowScvO_2 _events were defined as ScvO_2 _< 65% maintained for at least 5 minutes.

## Results

Thirty-seven patients (24 males) were enrolled. The mean clinical characteristics at admission to intensive care were: age 59 ± 16 years, BMI 26.1 ± 4.5 kg/m^2^, SAPS II 40 ± 13 (on 33 patients), PaO_2_/FiO_2 _206 ± 79, MAP 80 ± 13 mmHg, HR 92 ± 21 bpm, CVP 12 ± 3 mmHg, Hb 10.6 ± 1.9 g/dl. Continuous monitoring analysis detected 147 LowScvO_2 _events in 15 patients; while central venous blood gas analysis identified only nine LowScvO_2 _events in eight patients (6%). Table 1 summarizes patients' variables according to three ScvO_2 _ranges. SpO_2_, HR, MAP and CVP were not correlated with LowScvO_2 _events. Most patients had long periods of ScvO_2 _> 75 (supranormal ScvO_2_).

**Table 1:** Patients' variables according to ScvO_2 _range

	**ScvO**_**2 **_**< 65**	**ScvO**_**2 **_**65 to 75**	**ScvO**_**2 **_**> 75**
Patients	15/37	36/37	36/37
SpO_2 _(%)	95.8 ± 3.0	95.0 ± 3.3	96.4 ± 2.3
HR (bpm)	90.6 ± 16.1	90.5 ± 18.1	90.7 ± 16.5
MAP (mmHg)	82.5 ± 10.6	83.4 ± 12.7	82.2 ± 11.7
CVP (mmHg)	18.3 ± 4.6	20.2 ± 8.2	19.2 ± 5.5

## Conclusions

Continuous ScvO_2 _monitoring showed that most events of poor tissue oxygenation are relatively common, are not recognized by extemporary central venous blood gas analysis and are not mirrored by changes in SpO_2_, HR, MAP or CVP.

